# Methane Decomposition to Hydrogen Over Zirconia‐Supported Fe Catalysts–Effects of the Modified Support

**DOI:** 10.1002/open.202300112

**Published:** 2023-09-08

**Authors:** Mohammed Bayazed, Anis H. Fakeeha, Ahmed A. Ibrahim, Yousef M. Alanazi, Ahmed E. Abasaeed, Wasim U. Khan, Jehad K. Abu‐Dahrieh, Ahmed S. Al‐Fatesh

**Affiliations:** ^1^ Chemical Engineering Department College of Engineering King Saud University P.O. Box 800 Riyadh 11421 Saudi Arabia; ^2^ IRC Refining and Advanced Chemicals Research Institute King Fahd University of Petroleum and Minerals Dhahran 31261 Saudi Arabia; ^3^ School of Chemistry and Chemical Engineering Queen's University Belfast Belfast BT9 5AG Northern Ireland UK

**Keywords:** hydrogen generation, iron catalyst, methane decomposition, modified support, zirconia

## Abstract

Methane decomposition is a promising route to synthesize CO_x_‐free hydrogen and carbon nanomaterials (CNM_s_). In this work, the impregnation method was employed for the preparation of the catalysts. Systematic investigations on the activity and stability of Fe‐based catalysts were carried out in a packed‐bed micro‐activity reactor at 800 °C with a feed gas flow rate of 18 mL/min. The effect of doping Y_2_O_3_, MgO, SiO_2_ and TiO_2_ over ZrO_2_ on the catalytic performance was also studied. BET revealed that the specific surface areas and pore volumes are increased after SiO_2_, TiO_2_, and Y_2_O_3_ are added to ZrO_2_ while MgO had a negative impact and hence a little decrease in specific surface area is observed. The catalytic activity results showed that the Fe‐based catalyst supported over TiO_2_‐doped ZrO_2_ that is, Fe−TiZr, demonstrated the highest activity and stability, with a maximum methane conversion of 81.3 % during 180 min time‐on‐stream. At 800 °C, a maximum initial methane conversion of 73 %, 38 %, 64 %, and 69 % and a final carbon yield of 121 wt. %, 55 wt. %, 354 wt. %, and 174 wt. % was achieved using Fe−MgZr, Fe−SiZr, Fe−TiZr and Fe−YZr catalysts, respectively. Moreover, bulk deposition of uniform carbon nanotubes with a high degree of graphitization and different diameters was observed over the catalysts.

## Graphical abstract

Wet‐impregnated catalysts for the synthesis of hydrogen and carbon nanotubes from methane decomposition are reported in this article. The activity results in terms of methane conversion and carbon yield have shown that iron‐based catalysts supported on zirconia (Zr) modified with various oxide supports have demonstrated varying activity profiles. Catalysts supported on titania‐modified Zr remained the best‐performing catalyst.

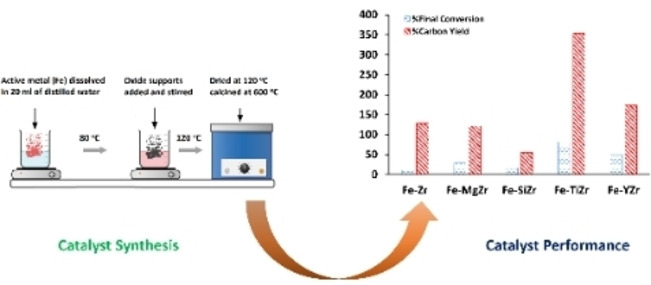

## Introduction

Hydrogen is an inspiring green energy alternative and can be used in many commercial applications (such as ammonia production, in refineries, and as a feedstock for chemicals). Since hydrogen produces more energy than any fossil fuel (per unit weight) and can replace fossil fuel.^[1]^ The difficulties posed by high CO_2_ emissions from methane‘s conventional conversion have led to the development of emission‐free hydrogen production from methane.^[2]^ Natural gas is often used to generate hydrogen via catalytic decomposition of methane (CDM), steam reforming of methane (SRM), partial oxidation of methane (POM), and dry reforming of methane (DRM).^[3]^ CDM is a simple and environmentally friendly process for producing hydrogen, due to its low energy consumption, high hydrogen purity, and single solid by‐product (carbon) formation.[^4^, ^5^, ^6^, ^7^]

Methane is the best source of hydrogen because it has the maximum ratio of hydrogen‐carbon in between all hydrocarbon, is more abundant than other fossil fuels, and as well as has a simpler conversion process.^[8]^ Methane can be decomposed into H_2_ and C in a simple reaction (Equation 1) as shown in the following^[9]^

(1) $${CH}_{4\left(g\right)}\to {C_{\left(s\right)}}_{\;}+{2H}_{2\left(g\right)}\;{\Delta H}_{298^{0}k}=74.8\;\;kJ/mol$$

At temperatures more than 550 °C, methane cracking is spontaneous and can happen even without the use of a catalyst.^[10]^ To achieve total breakdown, the reaction needs temperatures higher than 1000 °C.^[11]^ Nickel and cobalt catalysts are efficient for the CDM reaction at temperatures of 500 and 600 °C. However, these catalysts would deactivate quickly at higher temperatures. Ni and Co are also quite expensive and toxic. However, Fe catalysts are less expensive and non‐toxic, and it can be used with the produced nano carbon for a variety of applications without harming the environment.[^4^, ^12^] Although the Fe catalysts have a lower activity than Ni and Co‐based catalysts, the Fe catalysts can operate over wider temperature ranges, which may result in higher conversion. On the contrary, the catalytic activity performance of the active metals could be influenced by the textural promoters^[4]^ and support types used in the catalyst.^[13]^

CDM has been studied using silica‐supported Fe catalysts. A variety of Fe/SiO_2_ catalysts with Fe loadings ranging from 25 to 100 wt. % were created. Comparatively more activity was shown by all supported catalysts than by the unsupported 100 wt. % Fe. Additionally, as the Fe loading increased, the catalytic activity increased as well.^[14]^ reviewed the CMD and emphasized the need for industrial‐scale CDM in light of the current high CO_x_ emission levels and the advancement of Fe‐based catalysts. The most recent developments in the kinetic analysis of metal catalyst reactions are described, the effects of several parameters, like metal loading effect and support influences, on CDM catalyst performance,^[8]^ highlighted the need for a green alternative energy source because CH_4_ decomposition results in zero carbon emissions. They concentrate on the use of GAD reactors and discussed the impact of several parameters, including operation conditions, additives, geometric configuration, and catalysts on the performance.^[15]^

Zirconia‐based oxides offer acid/base properties, redox properties, and tunable phase composition that has attracted the interest of the scientific community. However, in the thermal decomposition of methane, there aren′t many studies that focus on the use of ZrO_2_‐supported catalysts with Fe and Ni oxides.^[16]^ Metal oxides like lanthanum, ceria, and zirconia were studied by Pudukudy et al.^[17]^ as catalyst support for Ni catalyst. High catalytic activity and stability were shown by all of the catalysts for methane decomposition. At 700 °C, lanthanum, ceria, and zirconia, supported catalysts produced an initial hydrogen yields of 58, 62, and 61 %, respectively, and final carbon yields of 1360 wt. %, 1159 wt. %, and 1576 wt. %. Metal oxides doping to zirconia is a strategy used to enhance a number of characteristics and the presence of the additive causes zirconia to crystallize in the tetragonal phase, which has a higher specific surface area as compared with the more stable monoclinic ZrO_2_. Al‐Fatesh et al.^[18]^ studied the impact of doping lanthanum (La_2_O_3_) and tungsten oxide (WO_3_) to ZrO_2_ on the performance of Fe‐containing catalysts. They found that the catalyst‘s stability was increased by the addition of La_2_O_3_, however, the use of WO_3_‐ZrO_2_ as support material enhanced its stability significantly. The catalyst with the label 20 wt. % Fe/WO_3_‐ZrO_2_ maintained its activity for 240 min that was assigned to the stronger metal‐support interaction, that is, interaction of Fe_2_O_3_ particles with the support (WO_3_‐ZrO_2_).

Silica is reported to prevent ZrO_2_ from crystallization at elevated temperatures that facilitates reservation of the higher specific surface area of the amorphous structure,^[19]^ whereas yttrium oxide with a valence lower than +4 favors the formation of oxygen vacancies, helping to store oxygen and return it at low temperatures.^[20]^ Additionally, it was shown that adding MgO to the ZrO_2_ support serves as a thermally stable catalyst support while effectively preserving the tetragonal ZrO_2_ phase.^[21]^ Gac et al.^[22]^ studied the effect of MgO addition on the activity of Ni/Al_2_O_3_ and found that the active surface area of Ni/Al_2_O_3_ increases as MgO is added to the system. The addition of MgO to the catalyst caused the growth of smaller nickel crystallites with more potent adsorption sites as a result of strong metal‐support interaction and the microstructure organization of the catalyst. The rate of methane decomposition increased during the initial stages of the reaction as the content of MgO increased.

TiO_2_ has poor thermal stability and a low specific surface area. In addition, under high‐temperature and high‐pressure conditions, the surface area of TiO_2_ would drastically decrease.^[23]^ Fortunately, it has been established by numerous researchers that doping titania with a different metal (such as Si, Fe, La, Zr, etc.) can effectively increase both its thermal stability and catalytic activity.[^24^, ^25^] Among various titania‐supported catalysts reported earlier,[^26^, ^27^] Fakeeha et al.^[26]^ examined the effect of various types of support materials, such as titania and magnesia on the performance of iron‐based catalysts. The results of the catalytic activity tests showed that all titanium‐supported catalysts exhibited less activity as well as deactivation. The Fe−Mg catalyst displayed the best activity (65 % conversion) for the duration of 3 h on the stream. TiO_2_−ZrO_2_ has attracted more attention as catalyst support because of the high specific area and high thermal stability compared to the relative single oxide. The addition of titanium to zirconium not only increases the specific surface area and thermal stability but also improves the acidity and basicity of zirconium and titanium.^[28]^

The objective of this study is to develop a Fe−Zr catalyst with high activity and stability for the catalytic decomposition of methane. For this purpose, the catalytic performance of Fe−Zr with different additives (Y_2_O_3_, MgO, SiO_2_, and TiO_2_) was developed and reported in this work. The fresh and spent catalysts are characterized to justify the findings using different techniques such as BET, H_2_‐TPR, O_2_‐TPO, XRD, TGA, and TEM.

## Results and Discussion

### Characterization of fresh catalysts

**Figure 1:**
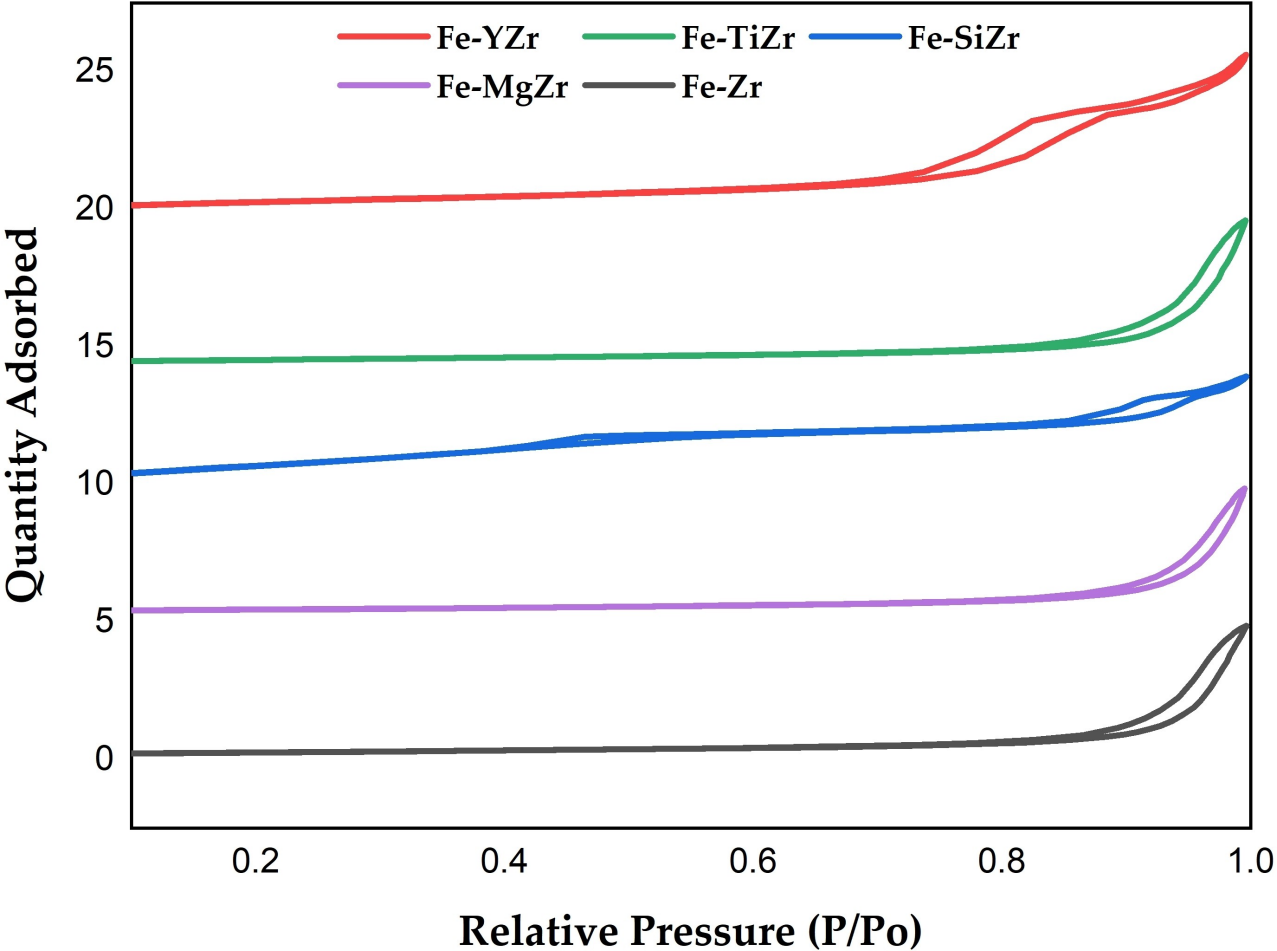
The textural properties of fresh supported Fe catalysts.

The compositions of the prepared catalysts were verified by EDX. For Instance, Figure 1S displays the spectra for 30 %Fe/10 %TiO_2_−ZrO_2_ catalyst. The result establishes that the prepared catalysts approximately assumed the desired composition as shown in Table 1S.

**Table 1:** Textural properties and description of the used catalysts.

Catalyst	Catalys Abbreviation	S.A. (m^2^/g)^[a]^	P.V. (cm^3^/g)^[a]^	P.S. (nm)^[a]^
30 %Fe/ZrO_2_	Fe−Zr	20	0.16	34.3
30 %Fe/10 %MgO−ZrO_2_	Fe−MgZr	19	0.16	36.3
30 %Fe/10 %SiO_2_−ZrO_2_	Fe−SiZr	127	0.176	5.4
30 %Fe/10 %TiO_2_−ZrO_2_	Fe−TiZr	23	0.176	33.4
30 %Fe/10 %Y_2_O_3_−ZrO_2_	Fe−YZr	51	0.218	14.9

[a] S.A.=surface area; P.V.=pore volume; P.S.=pore size, all evaluated from BET.

### Textural properties

The textural characteristics of the as‐synthesized catalysts were evaluated using N_2_ adsorption/desorption isotherms as shown in Figure 1. According to IUPAC sorting, the catalysts showed a type IV isotherm with a type H3 hysteresis loop, which is typical of a mesoporous structure with slit‐shaped pores of various sizes and shapes.^[29]^ As shown in Table 1, Fe−SiZr has a high surface area (127.2 m^2^/g) and an average pore size of 5.35 nm, which is beneficial in the metal additive‘s dispersion at high loading.^[30]^

Compared with Fe−Zr, the specific surface area and pore volume of Fe‐mZr (m=Si_,_ Ti, Y) is increased after SiO_2_, TiO_2_, and Y_2_O_3_ are added except Fe−MgZr that shows a little decrease. Nevertheless, the Fe−SiZr sample shows the highest surface area (127.2 m^2^/g) and lowest pore size (5.35 nm), while the Fe−MgZr sample displays the lowest surface (19.1 m^2^/g) and highest pore size (36.3 nm). The surface area of the unmodified Fe−Zr catalyst is 20.2 m^2^/g and remarkably increased to 22.8 m^2^/g and 50.9 m^2^/g for Fe−TiZr and Fe−YZr catalysts, respectively. Also, the pore volume of Fe−TiZr is found to be 0.173 cm^3^/g, which is higher than that of the unmodified Fe−Zr (0.157 cm^3^/g).

### X‐ray diffraction (XRD)

**Figure 2:**
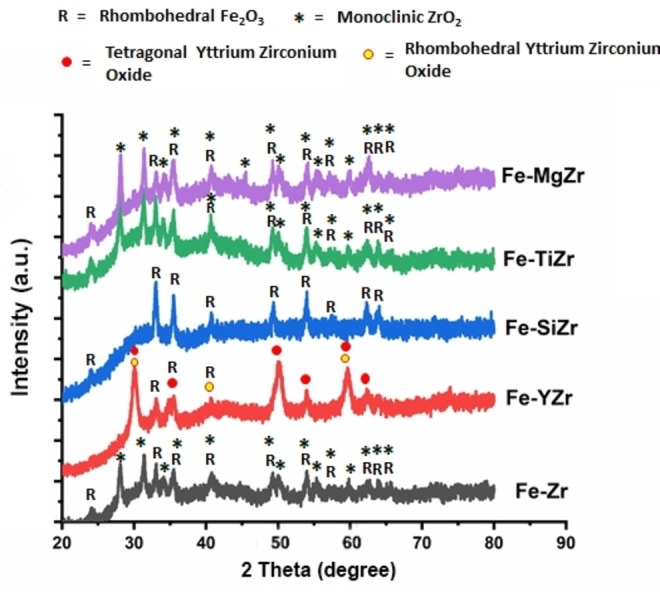
XRD patterns of zirconia‐supported Fe catalysts.

Powder XRD was conducted to study the crystal structures of the Fe‐loaded samples. The XRD patterns of zirconia‐supported Fe catalysts are presented in Figure 2. For Fe−Zr, Fe−MgZr and Fe−TiZr, the catalyst have only monoclinic ZrO_2_ phase (at Bragg's angle 2θ=24.01°, 28.10°, 31.40°, 34.09°, 35.46°, 40.74°, 49.28°, 50.11°, 54.04°, 55.35°, 57.11°, 59.87°, 62.75°, 64.03° and 65.72°; (Reference code 00‐037‐1484) and rhombohedral Fe_2_O_3_ phases (at Bragg's angle 2θ=24.01°, 33.04°, 35.46°, 40.74°, 49.28°, 54.04°, 62.75° and 64.03°; (Reference code 00‐024‐0072). Fe−SiZr catalyst has only rhombohedral Fe_2_O_3_ phases. Fe−YZr catalyst has rhombohedral Fe_2_O_3_ phases, tetragonal yttrium zirconium oxide phases (at Bragg's angle 2θ=30.10°, 35.57°, 50.15°, 54.03°, 59.74°, and 62.40°; (**Reference code 00‐048‐0224**) and Rhombohedral yttrium zirconium oxide phases (30.10°, 40.73°, and 59.74° (Reference code 00‐029‐1389). Typically, this behavior showed that the addition of Y_2_O_3_ had a significant impact on the phase composition of the zirconia support. Thus, we can infer that rather than the monoclinic ZrO_2_ (m‐ZrO_2_) phase, the growth of the tetragonal zirconium phases (t‐ZrO_2_) phase was enhanced by the addition of Y_2_O_3_. The degree of crystallinity of the zirconia‐supported Fe catalyst was calculated to be 12 %, 10.3 % and 10.2 % for the Fe−TiZr, Fe−YZr and Fe−Zr catalysts respectively. Moreover, the low intensity of the Iron peaks in the Fe−TiZr and Fe−YZr catalysts further confirms its fine surface dispersion on the catalyst support.

### H_2_‐TPR

**Figure 3:**
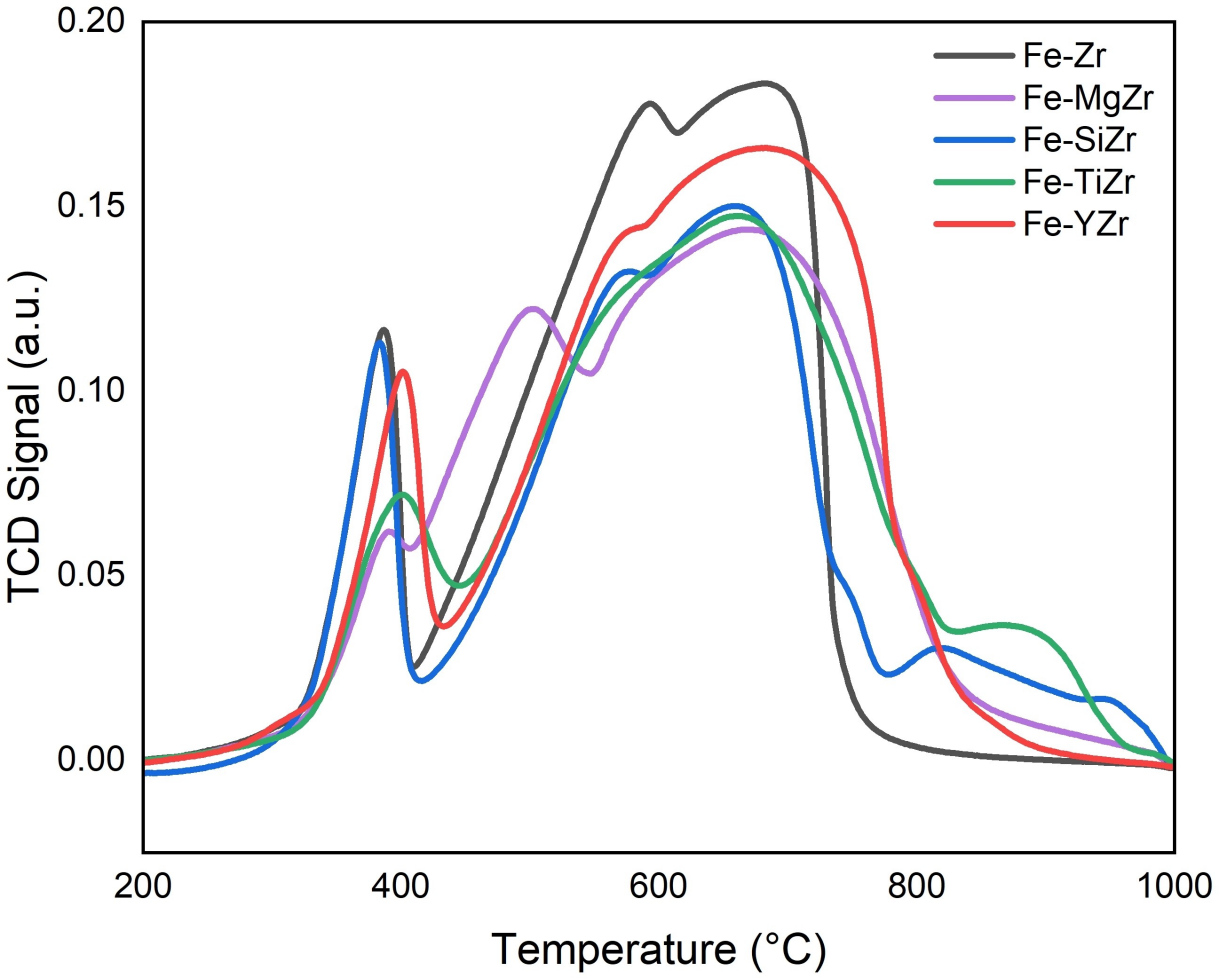
H_2_‐TPR of fresh supported Fe catalysts.

The H_2_‐TPR (Temperature‐programmed reduction) is an important technique for characterizing the catalyst‘s metal‐support interactions and reducibility. It may also be used to verify the catalyst‘s appropriate reduction temperatures.^[31]^ H_2_‐TPR curves of the fresh catalysts are shown in Figure 3. The peaks between 300 and 500 °C are ascribed to the reduction of Fe_2_O_3_ to Fe_3_O_4_. The unmodified and SiO_2_ modified samples showed identical profiles in terms of intensity and position, however, the rest of the modified samples displayed lower intensities, particularly MgO and TiO_2_ modified samples, and their peaks are slightly shifted to higher temperatures denoting stronger interactions. The broad peaks between 550 and 750 °C could be assigned to the further reductions of Fe_3_O_4_ to FeO, and Fe. The addition of modifiers diminished the intensities with respect to the unmodified sample. The peak for Y_2_O_3_ modified sample exhibited the highest intensity, while the rest of the modified samples exhibited similar intensities. At around 800 °C temperature, a weak H_2_ consumption peak appeared related to the Fe−TiZr sample. This could be attributed to the reduction of mixed oxide species to metallic species.^[18]^ This further enhances the dispersion of the active metals.

### TEM analysis

**Figure 4:**
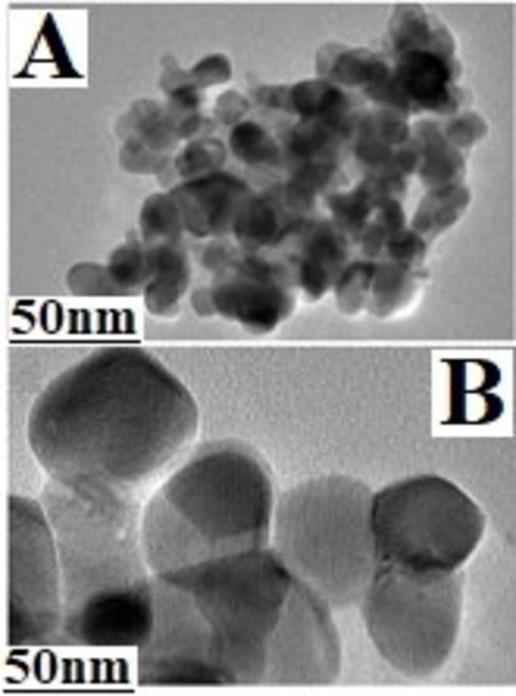
TEM images of the fresh Fe−TiZr catalyst.

The inner microstructure of the freshly calcined Fe−TiZr catalyst was investigated by TEM analysis and the images are shown in Figure 4. As illustrated in Figure 4a, the catalyst particles were observed to be closer to the form that is spherical and their boundaries are extremely clear. Besides, the dark spots shown in the TEM images represent the Fe_2_O_3_ particles were significantly smaller and much more separated, which further confirmed the greater dispersion associated with active Fe_2_O_3_ on surface of TiZr support (Figure 4a). The particle size of Fe_2_O_3_ nanoparticles was within the range of 24–32 nm. In addition, high‐resolution TEM images showed that Fe_2_O_3_ and ZrO_2_ particles exhibit well‐defined lattice spacing visible from the lattice edges of the catalyst particles (Figure 4b). These observations were very consistent with the XRD results.

### Catalyst activity

**Figure 5:**
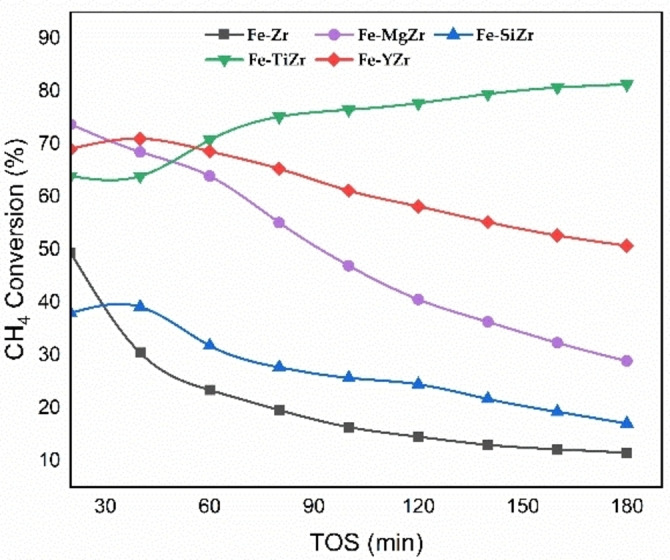
Methane conversion of fresh Fe‐supported catalysts at 800 °C.

The CDM reaction over Fe‐based catalysts was carried out at 800 °C under atmospheric pressure with a flow rate of 18 mL/min. The space velocity of the feed was maintained at 7200 mL h^−1^ g_c_^−1^. Figure 5 shows the effect of different additives (Y_2_O_3_, MgO, SiO_2_, and TiO_2_) on the methane conversion for 180 min time on‐stream (TOS) using 30 wt. % Fe supported on ZrO_2_ catalysts. The results exhibited that the Fe−TiZr sample gave the highest conversions of methane at about 81.3 % and kept increasing over the period of the investigation (3 h). In contrast, Fe−MgZr, Fe−SiZr, and Fe−Zr catalysts began with initial CH_4_ conversions of about 74 %, 38 % and 50 % respectively, but suffered fast deactivation to attain as low values as 29 %,17 % and 11.5 %, respectively after running 180 min of TOS (Figure 5).

A high methane conversion of about 69 % was achieved in the Fe−YZr catalyst at the temperature of 800 °C. The addition of yttria (Y_2_O_3_) in the catalyst structure led to an increase in its catalytic activity. However, its activity decreased with time to about 50.1 % due to carbon deposition on active Fe surfaces.^[32]^

The catalytic activity of Fe−Zr improved significantly by adding Titanium and the methane conversion of Fe−TiZr increased from about 64 % to 81.3 % after 180 min time on‐stream. The high dispersion of Fe_2_O_3_ particles, which resulted from their proper interaction with the TiZr support, may be primarily responsible for the Fe−TiZr catalyst‘s pronounced catalytic activity and durability. The addition of Titanium could also accelerate the growth of carbon fiber or carbon nanotubes and stop the formation of amorphous carbon over active sites.^[31]^ Consequently, the Fe−TiZr catalyst‘s lifetime was prolonged, due to the availability of active sites with Fe−Zr.

The low methane conversion and hydrogen yield at the beginning of the reaction of Fe−TiZr can be attributed to the in‐situ consumption of H_2_ released during the decomposition of methane, which continued the reduction of unreduced Fe active sites left over from the pre‐reduction step. This in‐situ reduction increases the total number of active sites available for the reaction, which in turn promotes methane decomposition and carbon formation until equilibrium is reached.[^33^, ^34^]

From the results, it can be inferred that among all the dopants used with ZrO_2_, TiO_2_ enhanced the catalyst's performance. On the other hand, the weak interaction between the catalyst‘s components can be used to explain the low catalytic activity and stability of other zirconia‐supported catalysts. This behaviour causes a metal sintering that produces large metal particles, especially with higher calcination temperatures.^[18]^

The performance comparison between the current work and others in the literature is summarized in Table 2. Furthermore, in contrast to Ref. [10], where 20 % Fe was sustained with Zr stabilized with La and W, in this work 30 % Fe was supported with Zr stabilized with Ti, Mg, Si, and Y. Alternatively, in Ref. [23], the CMD was performed using promoted 60 % Ni catalyst supported only by Al_2_O_3_.

**Table 2:** Comparison of the catalytic performance of Fe‐based catalysts with previously reported results.^[a]^

Catalysts	T_R_ [°C]	TOS [min]	CH_4_ Conv. [%]	Ref.
20 %Fe/ZrO_2_	800	240	60	[18]
20 %Fe/La_2_O_3_+ZrO_2_	800	240	80	[18]
20 %Fe/WO_3_+ZrO_2_	800	240	91	[18]
75 %Fe/SiO_2_	650	400	43	[14]
50 %Fe/Y_2_O_3_	800	240	34	[35]
27 %Fe/La_2_O_3_	800	360	33	[36]
25NiCo/TiO_2_	700	300	83	[27]
50 %Fe/MgO	700	570	45	[37]
Fe/Al/Si ratio (wt %: 20 : 80)	800	300	64	[38]
Fe/Al/Si ratio (wt %: 100 : 0)	800	300	83	[38]
30 %Fe/TiO_2_+ZrO_2_	800	180	82	This work
30 %Fe/Y_2_O_3_+ZrO_2_	800	180	69	

[a] T_R_=Reaction Temperature.; TOS=Time on stream; CH_4_ Conv.=methane conversion.

### Characterization of spent catalysts

#### Temperature programmed oxidation (TPO)

**Figure 6:**
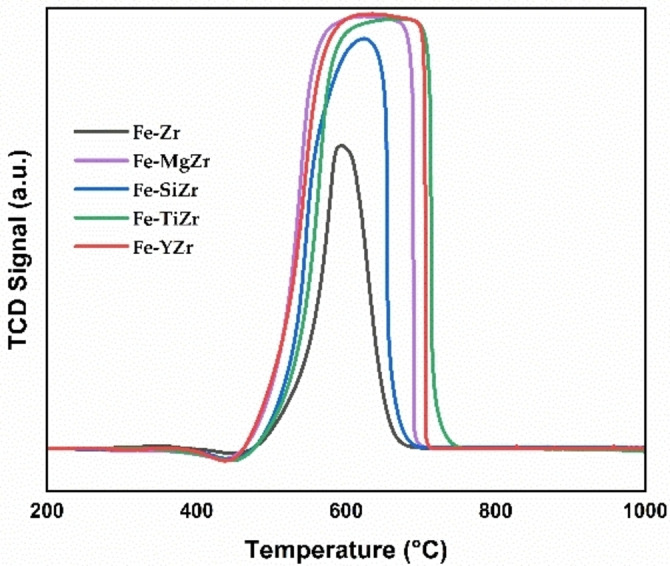
TPO profiles of the spent supported Fe catalysts.

TPO is one of the useful methods that can be used to identify the type of carbon deposited onto the surface of used catalysts and it has also been performed to evaluate the spent catalysts′ thermal stability (Figure 6). From the profile curves, it is first observed that there was a slight gain in the mass at around 400 °C, which can be attributed to the oxidation of the metallic species present in the catalysts, followed by a broad gain in mass between 500 and 700 °C. The low‐temperature peak (<400 °C) corresponds to the coke‐like carbon (amorphous carbon) which covers the active metal species resulting in catalyst deactivation. The high‐temperature peak (500–700 °C) corresponds to the highly ordered carbon nanostructures which have the crystalline graphitic structures.[^39^, ^40^] The result is indicating a highly ordered crystal structure (600–750 °C) with the absence of amorphous carbon (~400 °C). For Fe−TiZr catalyst, the TPO curve (Figure 6) had a weight loss peak at ~700 °C where the highest temperature peak was dominant. The increase in the amount of carbon deposited by titania doping agreed with the result obtained in the stability test after 180 min of on‐stream reaction at 800 °C, which means that more amounts of hydrogen and carbon were produced from methane decomposition.^[41]^

#### TGA

**Figure 7:**
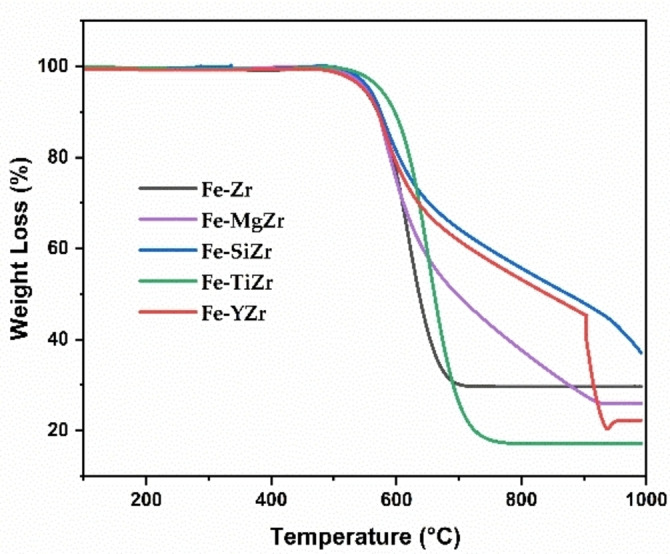
The weight loss of the carbon deposits over zirconia‐supported Fe catalysts.

**Figure 8:**
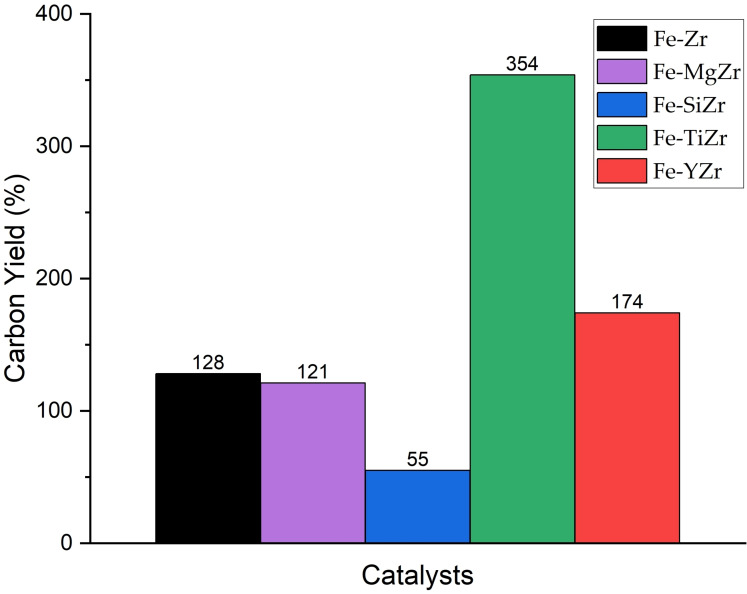
Carbon yield as a function of mixed metal oxide catalysts composition.

The amount of deposited carbon is frequently calculated using thermogravimetric analysis (TGA).^[42]^ The weight loss of the carbon deposits over zirconia‐supported Fe catalysts was shown in Figure 7. The total carbon accumulation on the spent catalysts was expressed as weight loss percentages. Different types of carbon oxidize at different temperatures, and the combustion of amorphous carbon is primarily responsible for weight loss in TGA curves below 450 °C.^[43]^ At 800°C reaction temperature, the catalyst Fe−TiZr exhibited the highest weight loss of 83 %, followed by Fe−YZr_,_ Fe−MgZr and Fe−Zr catalysts that presented 78 %, 74 % and 71 % weight loss respectively, and ultimately Fe−SiZr catalyst displayed the lowest carbon weight loss of 63 % and hence the minimum carbon formation on it. In addition, the carbon yield of 121 wt. %, 55 wt. %, 354 wt. %, and 174 wt. % was achieved using MgO, SiO_2_, TiO_2_, and Y_2_O_3_ modified over zirconia‐supported catalysts respectively (Figure 8). The weight loss peak observed in the Fe−YZr sample at around 900 °C is likely due to the weight gain resulting from the oxidation of residual Fe, which takes place after the oxidation of all carbon deposits.^[33]^ The Fe−TiZr catalyst showed the highest weight loss of deposited carbon, which was in good agreement with the catalyst‘s high activity. These findings infer that the TiO_2_ addition promoted the efficiency of the Fe/ZrO_2_ catalyst and thus increased the accumulation of carbon on the catalyst surface.

#### Raman spectra

**Figure 9:**
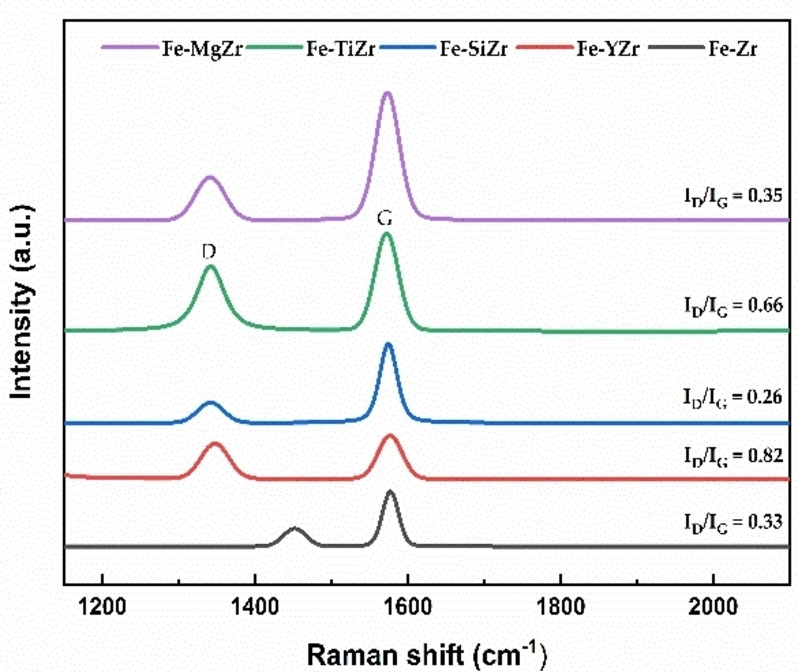
Raman spectra of the used catalysts.

Raman spectra of the used catalysts are presented in Figure 9. All the spent catalysts displayed D, G band peaks. The D band peaks at around 1350 cm^−1^ is assigned to the disordered carbon, including amorphous carbon or defective graphite sheets. On the contrary, the G band peaks at around 1580 cm^−1^ correspond to C−C stretching vibrations that are characteristic of graphite.^[44]^ The integral intensity ratio I_D_/I_G_ is widely used to express the degree of graphitization for the carbon, that is, the lower the I_D_/I_G_ ratio, the higher the crystalline order of the carbon species. The I_D_/I_G_ values of all samples means that the carbon is ordered, with minor contributions from disordered particles.^[45]^ As seen from Figure 9, the values of I_D_/I_G_ are different, indicating that the deposited carbon on Fe−TiZr and Fe−YZr catalysts had less graphitization degree than other zirconia‐supported Fe catalysts, which is in accordance with the formation of more carbon on the surface of Fe−TiZr catalysts.

#### SEM and TEM study

**Figure 10:**
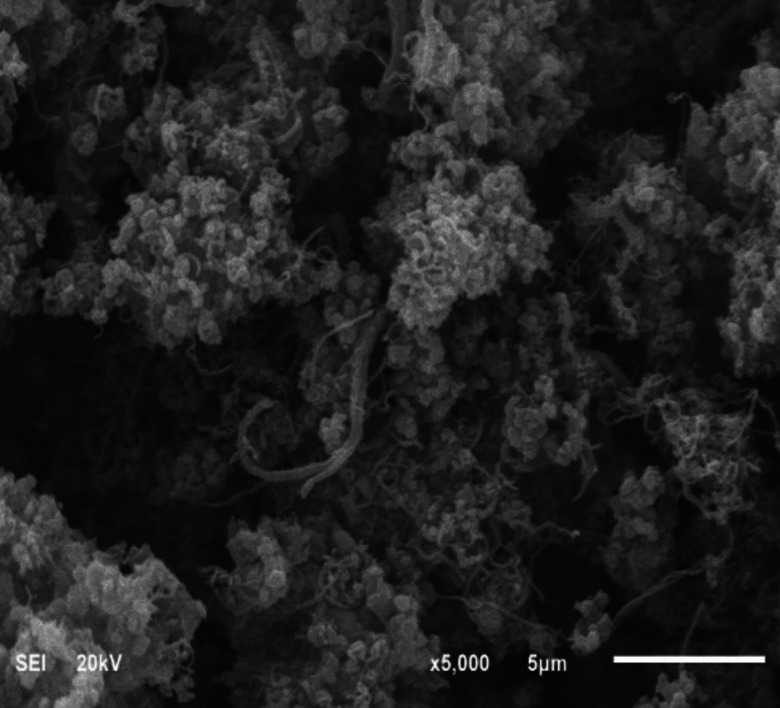
SEM images of the nanocarbon deposited over Fe−TiZr catalyst.

**Figure 11:**
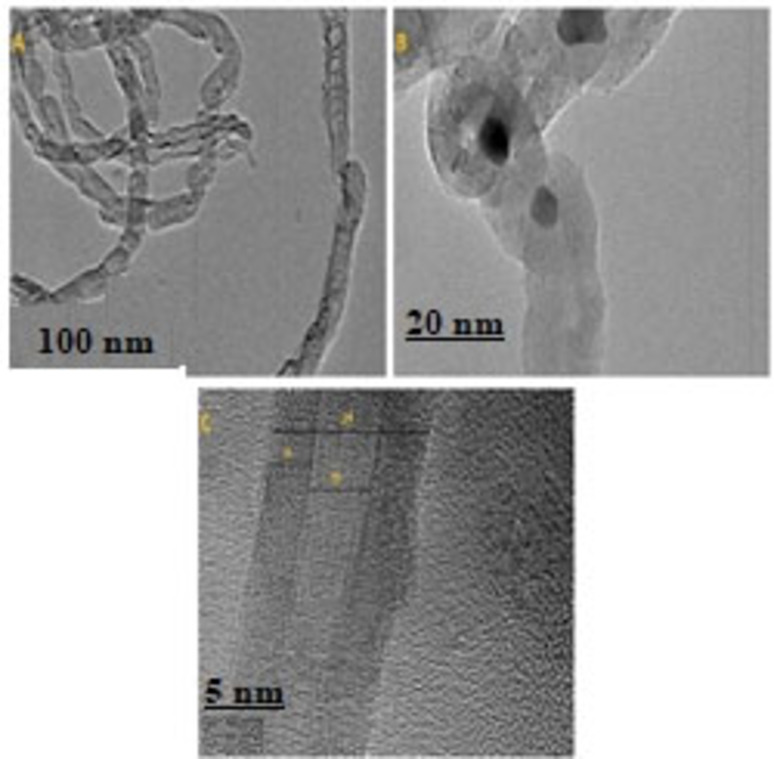
TEM images of the nanocarbon deposited over Fe−TiZr catalyst.

The microstructural changes and morphology of the deposited carbon over the most active Fe−TiZr catalyst at 800 °C reaction temperature and 3 h time on stream were studied by SEM and TEM analysis and the images are shown in Figures 10 and 11, respectively. SEM image of Fe−TiZr catalyst indicates the deposition of filamentous carbon during methane decomposition reaction. It is obvious that carbon nanomaterials with various aspect ratio are formed over Fe−TiZr. Due to intermingled nanomaterials, it is challenging to identify the exact length and/or diameter of nanomaterials however, some of the nanomaterials were of millimeter range. As illustrated in Figure 11a, the deposited carbon material on the surface of Fe−TiZr was multi‐walled carbon nanotubes (MWCNTs) with different diameters. A mixture of large‐diameter MWCNTs with hollow and full cores was obtained, Moreover, the MWCNTs have some defects in the outer walls (Figure 11a). ^[18]^ The dark spots shown in Figure 11b represent the metallic Fe nanoparticles with spherical shapes that were clearly observed inside the hollow core of MWCNTs. The high‐resolution TEM image in Figure 11c, further confirms the presence of MWCNTs with a clear hollow core, where the diameter of 25.5 nm, wall thickness of 8 nm and an internal channel space of 10 nm was observed.^[46]^

## Conclusions

This work reports wet‐impregnated Fe‐based zirconia‐supported catalysts for catalytic decomposition of methane. Zirconia was modified by doping with metal oxides including MgO, SiO_2_, TiO_2_, and Y_2_O_3_. From the results of catalyst activity, TiO_2_−ZrO_2_ incorporated is found to be an excellent support for Fe‐based catalyst towards CH_4_ decomposition reaction on time on stream, the catalytic performance of the Fe−MgZr catalyst drops fast than Fe−YZr catalyst. Even after 180 min, CH_4_ conversion over Fe−YZr catalyst does not drop below 50 %. It is also found that the Fe−TiZr catalyst was more stable than other zirconia‐supported catalysts and it could be responsible for the highest carbon yield over the Fe−TiZr catalyst, even it showed low surface area. The high stability of the titanium‐supported catalyst could be attributed to the formation of nanosized active Fe and its fine dispersion on the titanium zirconia matrix after the reduction of spinel solid solution.

## Experimental Section

### Materials

Zirconium oxide and other oxide used were obtained from DAIICHI KIGENSO KAGAKU KOGYO CO., LTD. OSAKA – JAPAN. The precursor for iron was ferric nitrate nonahydrate (Fe (NO_3_)_3_ ⋅ 9H_2_O, purity 99 %, laboratory reagents & fine chemicals). Millipore system of water purification was used to obtain ultrapure water.

### Catalysts preparation

**Figure 12:**
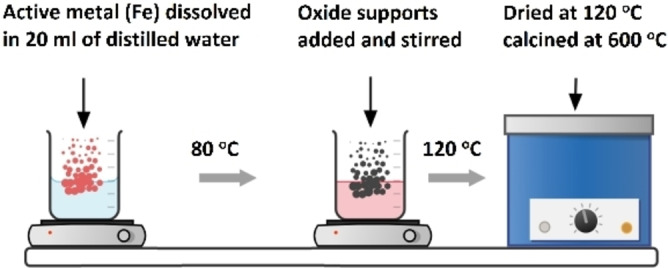
The scheme of the catalyst preparation.

The wetness impregnation route was chosen to synthesize iron‐based catalysts supported over ZrO_2_ (Figure 12). The active metal loading of iron was fixed at 30 wt. %. Firstly, stoichiometric quantity of active metal precursor was dissolved in 20 mL of double‐distilled water followed by addition of oxide supports. Subsequently the mixture was subjected to magnetic stirring for 3 h at 80 °C. Later, the drying of slurry samples was done using a furnace at 120 °C for 16 h. The final step was to calcine the samples at 600 °C for 3 h.

### Catalytic activity test

The testing of as‐synthesized catalysts was conducted at a reaction temperature of 800 °C at 1 bar pressure using a fixed‐bed tubular reactor purchased from PID Eng & Tech. Firstly, glass wool was placed into the reactor to serve as the catalyst bed over which 0.15 g of catalyst was loaded. A K‐type thermocouple was axially located in the reactor to measure the temperature of the catalyst bed during CDM. The fresh catalyst was reduced under hydrogen for 1 h at 800 °C prior to flowing reaction gas. The gaseous feedstock containing 12 mL/min of CH_4_ and 6 mL/min of N_2_ was used for CDM investigation. The reactor outlet was analysed using an online Gas Chromatography (GC‐Shimadzu 2014) equipped with a thermal conductivity detector. The conversion of methane (X_CH4_) and Carbon yield were calculated using following Equations (2) and 3:

(2) $$X_{CH4}\;\%=\frac{\left[{CH}_{4,in}\right]\;-\;{[CH}_{4,out}]\;}{\left[{CH}_{4,in}\right]}\;\times 100$$

(3) $$Carbon\;yield\;\%=\frac{\left[W_{P}\right]-\;\left[W_{cat}\right]}{\left[W_{cat}\right]}\;\times 100$$

Where ${CH}_{4,in}$ is methane in the feed, ${CH}_{4,out}$ is methane in the product, W_P_ is the products weight after reaction and W_cat_ is the weight of the fresh catalyst.

### Catalyst characterization

The textural properties of the as‐synthesized catalysts were measured using a Micromeritics Tristar II 3020 analyser at 77 K. Prior to porosity analysis, drying/degassing of each catalyst was conducted at 250 °C for 3 h under nitrogen to remove any surface impurities. Barret‐Joyner‐Halenda (BJH) method was utilized for evaluating the pore volume of the catalysts.

The chemisorption equipment purchased from Micromeritics (Auto Chem II 2920) was utilized for temperature‐programmed reduction (TPR) and oxidation (TPO). The catalysts were subjected to temperatures variation between 50 and 900 °C under a mixture of 10 % hydrogen in argon flowing at 2.40 L/h for the TPR analysis while 10 % hydrogen in argon was replaced with 10 % oxygen in helium for TPO analysis of type of carbon formed during CDM.

X‐ray diffraction profiles of as‐synthesized catalysts were measured using a Miniflex Rigaku diffractometer containing Cu K as source of X‐rays. The diffractometer was operated at 40 mA and 40 kV.

Laser Raman (NMR‐4500) Spectrometer (JASCO, Japan) was used to evaluate the type of carbon formed during CDM. The spectrometer used a 532 nm wavelength excitation beam with an objective lens capable of 100×magnification as well as 1.6 mW of laser intensity. Three exposures within 10 seconds were averaged to collect each spectrum. Spectrometer recordings within Raman shift of 1200–3000 cm^−1^ were later processed via Spectra Manager Ver. 2 software.

The quantity of carbon deposits on the spent catalysts was measured using TGA analysis. In a typical analysis, 10–15 mg of the spent catalysts was subjected to thermal variation ranging from 50 to 1000 °C at a ramp rate of 20 °C/min under oxidative environment. Finally, the weight loss with respect to temperature was monitored.

## Supporting Information

The Supporting Information contains Figure S1, an EDX image of 30 %Fe/10 %TiO_2_−ZrO_2_ catalyst and Table S1, showing quantitative result of the 30 %Fe/10 % TiO_2_−ZrO_2_ catalyst.

## Conflict of interest

The authors declare no conflict of interest.

1

## Supporting information

As a service to our authors and readers, this journal provides supporting information supplied by the authors. Such materials are peer reviewed and may be re‐organized for online delivery, but are not copy‐edited or typeset. Technical support issues arising from supporting information (other than missing files) should be addressed to the authors.

Supporting Information

## Data Availability

The data that support the findings of this study are available from the corresponding author upon reasonable request.
