# Supplementary material for: Methane Decomposition to Hydrogen Over Zirconia‐Supported Fe Catalysts–Effects of the Modified Support
Source: ChemistryOpen. 2023 Sep 8;12(9):e202300112. doi: 10.1002/open.202300112 (PMC10491930; doi:10.1002/open.202300112)
Supplement: Supplementary file 1 — Supporting Information [file OPEN-12-e202300112-s001.pdf]

ChemistryOpen

Supporting Information

Methane Decomposition to Hydrogen Over Zirconia-Supported Fe Catalysts–Effects of the Modified Support

Mohammed Bayazed, Anis H. Fakeeha, Ahmed A. Ibrahim, Yousef M. Alanazi, Ahmed E. Abasaeed, Wasim U. Khan, Jehad K. Abu-Dahrieh,* and Ahmed S. Al-Fatesh*

The Supporting Information contains:

- Figure S1 EDX image of 30%Fe/10%TiO_2 - ZrO_2 catalyst.
- Table S1 Quantitative result of 30%Fe/10%TiO_2- ZrO_2 catalyst.

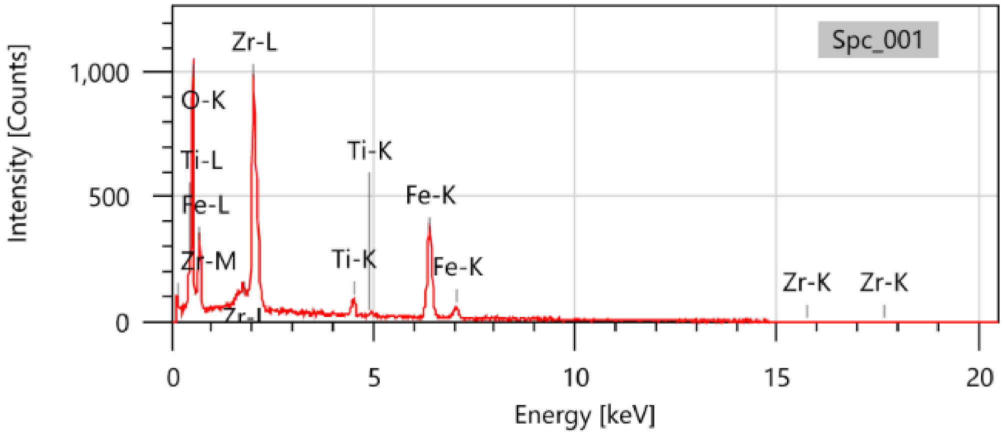

Figure S1 EDX image of 30%Fe/10%TiO_2 - ZrO_2 catalyst.

Table S1 Quantitative result of 30%Fe/10%TiO_2- ZrO_2 catalyst.

**Display name**	**Standard data**	**Quantification method**	**Result Type**
Spc_001	Standardless	ZAF	Metal
Element	Line	Mass %	Atom%
O	K	24.63±0.30	57.92±0.70
Ti	K	7.30±0.13	6.59±0.10
Fe	K	33.40±0.56	21.19±0.38
Zr	L	34.67±0.44	14.30±0.18
Total		100	100
Spc_001			Fitting ratio 0.0779
